# Role of the Pro-Inflammatory Tumor Microenvironment in Extracellular Vesicle-Mediated Transfer of Therapy Resistance

**DOI:** 10.3389/fonc.2022.897205

**Published:** 2022-05-11

**Authors:** Layla Simón, Sofía Sanhueza, Belén Gaete-Ramírez, Manuel Varas-Godoy, Andrew F. G. Quest

**Affiliations:** ^1^Laboratory of Cellular Communication, Program of Cell and Molecular Biology, Center for Studies on Exercise, Metabolism and Cancer (CEMC), Institute of Biomedical Sciences (ICBM), Faculty of Medicine, University of Chile, Santiago, Chile; ^2^Advanced Center for Chronic Diseases (ACCDiS), Faculty of Medicine, Universidad de Chile, Santiago, Chile; ^3^Escuela de Nutrición y Dietética, Universidad Finis Terrae, Santiago, Chile; ^4^Cancer Cell Biology Laboratory, Centro de Biología Celular y Biomedicina (CEBICEM), Facultad de Medicina y Ciencia, Universidad San Sebastián, Santiago, Chile; ^5^Centro Ciencia & Vida, Fundación Ciencia & Vida, Santiago, Chile

**Keywords:** extracellular vesicles, exosomes, inflammation, therapy resistance, tumor microenvironment

## Abstract

Advances in our understanding of cancer biology have contributed to generating different treatments to improve the survival of cancer patients. However, although initially most of the therapies are effective, relapse and recurrence occur in a large percentage of these cases after the treatment, and patients then die subsequently due to the development of therapy resistance in residual cancer cells. A large spectrum of molecular and cellular mechanisms have been identified as important contributors to therapy resistance, and more recently the inflammatory tumor microenvironment (TME) has been ascribed an important function as a source of signals generated by the TME that modulate cellular processes in the tumor cells, such as to favor the acquisition of therapy resistance. Currently, extracellular vesicles (EVs) are considered one of the main means of communication between cells of the TME and have emerged as crucial modulators of cancer drug resistance. Important in this context is, also, the inflammatory TME that can be caused by several conditions, including hypoxia and following chemotherapy, among others. These inflammatory conditions modulate the release and composition of EVs within the TME, which in turn alters the responses of the tumor cells to cancer therapies. The TME has been ascribed an important function as a source of signals that modulate cellular processes in the tumor cells, such as to favor the acquisition of therapy resistance. Although generally the main cellular components considered to participate in generating a pro-inflammatory TME are from the immune system (for instance, macrophages), more recently other types of cells of the TME have also been shown to participate in this process, including adipocytes, cancer-associated fibroblasts, endothelial cells, cancer stem cells, as well as the tumor cells. In this review, we focus on summarizing available information relating to the impact of a pro-inflammatory tumor microenvironment on the release of EVs derived from both cancer cells and cells of the TME, and how these EVs contribute to resistance to cancer therapies.

## Introduction

Due to its high prevalence and mortality, cancer is now considered as the leading cause of death worldwide as defined by the World Health Organization (WHO) in 2019 (1). This multifactorial disease is characterized by the presence of cells that constantly proliferate in a rapid and uncontrolled manner (2). Currently, several methods exist for the treatment of cancer, including radiation therapy, surgery, immunotherapy, endocrine therapy, gene therapy and chemotherapy, the latter being the most commonly employed therapeutic approach (3).

While cancer treatments are initially quite successful, the long-term of success of such interventions is often limited by the development of drug resistance. As an example, in chemotherapy, 90% of cancer patient mortality is attributable to drug resistance (3). Processes leading to resistance can be segregated into two major categories, referred to as intrinsic or extrinsic, depending on whether the resistance was pre-existing in cancer cells, or subsequently acquired in response to treatment, respectively (4). Nevertheless, both types of resistance share common mechanisms that permit escaping cancer therapy, such as enhanced drug efflux, changes in drug targets, metabolic adaptations, dysregulation of the DNA damage repair machinery, defective apoptotic signaling, activation of pro-survival signaling, and other adaptive cellular responses (3, 5, 6).

Solid tumors display great cell heterogeneity and, together with non-cellular components, are referred to as the tumor microenvironment (TME) (7). The bidirectional communication between tumor cells and the surrounding stromal components plays a critical role in the regulation of tumor progression by favoring processes, such as metastasis and therapeutic resistance (8). The TME consists of non-cellular components, such as the extracellular matrix, and stromal cells, including cancer-associated fibroblasts (CAFs), mesenchymal cells, endothelial cells, adipocytes, and immune cells like the tumor associated macrophages (TAMs) (8). The TME is described as a pro-inflammatory microenvironment given that many of the cells present are inflammatory cells, and many cells of the TME have the ability to secrete pro-inflammatory molecules in response to different conditions including, but not limited to, hypoxia or chemotherapy (9–13). Pro-inflammatory processes also contribute to tumor progression, making the ability to suppress such events highly desirable for the successful outcome of treatments (9, 14). Thus, although cancer therapy has focused for many years primarily on tumor cells as the targets, the importance of the TME and interactions between tumor cells and the stromal components in promoting tumor development and progression, makes targeting these interactions an increasingly interesting option for cancer treatment (7, 15).

Intercellular communication in the TME is mediated by soluble factors such as cytokines, chemokines, growth factors, and extracellular vesicles (EVs) (16). EVs are a heterogeneous group of cell-derived membranous structures that are released to the extracellular space and are involved in multiple physiological and pathological processes, given that they represent vehicles for the transfer of a large variety of molecules to recipient cells, including DNAs, mRNAs, proteins, microRNAs (miRNA), long non-coding RNAs (LncRNAs), lipids, and metabolites. These days, the release and uptake of EVs is considered an important mechanism of intercellular communication and EVs are classified into two main groups according to their origin, namely exosomes that are of endosomal origin (30–150 nm in diameter), and microvesicles that are liberated directly from the plasma membrane (MVs, 50–500 nm in diameter), including apoptotic bodies (17). The content of the EVs is decisive in determining the phenotypic changes that may be triggered in recipient cells, and this in turn depends on the origin and the state of the cell when the vesicles are generated (18). For instance, EVs control several physiologically important functions such as immune surveillance, blood coagulation, stem cell maintenance and tissue repair. On the other hand, in some contexts, EVs have a pathological role. For example, EVs can favor the development of cancer, autoimmune diseases, prion diseases, neurodegeneration and HIV infection (19). Furthermore, EVs have been implicated in the acquisition of the hallmarks of cancer and driving tumor progression by promoting communication between cancer cells and the tumor microenvironment (20).

To contextualize the concept of EVs, the International Society for Extracellular Vesicles (ISEV) suggests minimal requirements to define vesicles as EVs (21). In general, EVs are structures with a lipid bilayer that are unable to replicate and lack a functional nucleus. In terms of specific markers, there is no consensus that permits clearly defining EVs of endosomal origin (exosomes) or those derived from the plasma membrane (ectosomes, microparticles, or microvesicles). Moreover, experimental limitations generally do not allow separating the different EV subpopulations. However, the ISEV recommends the use of size to define such subpopulations, and following those guidelines they can be separated into two main groups, small EVs (sEVs) (<200 nm in diameter, and medium/large EVs (m/lEVs) (>200 nm in diameter). Besides size, EVs also should be characterized by the presence of at least three positive protein markers of EVs and one negative marker to evaluate contamination by vesicles from other subcellular compartments. If an EV preparation does not meet these minimal requirements, the use of the term extracellular particles (EPs) is recommended. Therefore, the processing of samples, depending on the source of the EVs (conditioned medium or biological fluids), the experimental conditions (hypoxia or serum concentration for example), and the methods used to separate and concentrate the EVs (ultracentrifugation, size exclusion chromatography, among others) are crucial to achieve the minimal requirements to obtain vesicles considered as EVs. In this context, there are several methods to separate and concentrate EVs, but each one is different in terms of recovery and specificity. Therefore, to evaluate a biological effect of EVs, such as transfer of therapy resistance, it is important to consider which method is used. In this review, we summarize the main results of several articles which isolate, characterize and describe the role of vesicles in therapy resistance. In most, but not all cases these can be defined as EVs by the aforementioned criteria.

The important role of EVs in the communication between cancer cells and the TME, and their contribution to the development of different hallmarks that drive tumor progression is well established (20). Moreover, the biogenesis of EVs and their content are modulated by the different stimuli and conditions present in the TME. In this context of note is the ability of pro-inflammatory conditions to promote the release of EVs, which endow cancer cells with traits that permit developing resistance to anti-cancer therapies (22). With this in mind, we will focus in this review on summarizing how the pro-inflammatory tumor microenvironment and EVs generated in this milieu contribute to the acquisition of cancer therapy resistance.

## EVs in Cancer Therapy Resistance Induced by Hypoxia and Glycolysis

Hypoxia generates a pro-inflammatory TME that promotes resistance to cancer therapy (23). Several cell types are affected by hypoxic conditions that promote tumor cell survival, migration, invasion, and metastasis (24). Glycolysis appears as an important mechanism in this context. Indeed, a well-established hallmark of cancer that enhances tumor cell aggressiveness is metabolic reprogramming (25). Cancer cells impair mitochondrial respiration and convert to a glycolytic metabolism to obtain energy and intermediate metabolites required for tumor growth and metastasis (26). Consistent with the relevance of this switch, some drugs that prevent hypoxia-induced therapy resistance, like dichloroacetate, wogonin and baicalein, also inhibit glycolytic enzymes such as HKII, PDHK1, and LDHA (27–29). Moreover, inhibiting glucose uptake or the glycolytic pathway prevents hypoxia-induced therapy resistance (27, 30) due to HIF-1α downregulation mediated by the PTEN/PI3K/Akt/mTOR signaling pathway (28, 29, 31).

On the other hand, there is evidence suggesting that hypoxia-induced therapy resistance is independent of HIF-1α (32). Indeed, STAT3, rather than HIF-1α, appears as a key regulator in this process (33, 34). Circular RNA AKT3 (CircAKT3) is upregulated in cancer and inhibits miR-516b-5p, an inhibitor of STAT3, thereby promoting STAT3 activation and therapy resistance (35). One of the effects of the STAT3 activation is the downregulation of PTEN (36). Indeed, some authors have observed that activation of STAT3/Akt/MAP2K and PKM2/glycolysis are relevant in drug-resistant cells (35, 37). In addition to these cell intrinsic pathways, it has more recently become clear that cell extrinsic events involving EVs are important in events leading to therapy resistance.

Since hypoxia promotes EV production, several studies suggest that the hypoxia-related effects may be dependent on the delivery of proteins and nucleic acids present in EVs, which induce therapy resistance in recipient cells. Indeed, therapy-resistant cells are known to deliver EVs to therapy-sensitive cells and induce therapy resistance under hypoxic conditions (**Supplementary Table 1**). For instance, ovarian cancer cells exposed to hypoxia increase EV release by upregulating Rab27a and downregulating Rab7, LAMP1/2 and NEU-1. In this way, cisplatin-resistant cells deliver EVs containing STAT3 and FAS to sensitive cells and promote invasion through MMP2 expression and chemotherapy resistance under hypoxic conditions (38). Another mechanism observed in cancer cells exposed to hypoxia is the release of PKM2-containing EVs, which promote therapy resistance by stimulating glycolysis, ROS production and inhibiting apoptosis (39). In addition, EVs from oxaliplatin-resistant cancer cells deliver circR-122 to drug-sensitive cells. Here, circR-122 acts as a sponge for miR-122, the inhibitor of PKM2, thereby promoting PKM2 expression, glycolysis, and therapy-resistance (40). Moreover, other glycolytic enzymes, such as ALDOA and ALDH3A1, are detected in EVs of radiation-resistant cells. The transfer of these enzymes promotes glycolysis and aggressiveness in recipient cells (41).

HSP70 and Osteopontin are stress proteins that participate in hypoxia-induced radio- and chemotherapy resistance. HSP70 is present at the plasma membrane and naturally released in EVs. As hypoxia stimulates EV production, an increment in HSP70 levels in plasma is observed that promotes therapy resistance. Osteopontin expression also increases under hypoxic conditions. In fact, increases in HSP70 and Osteopontin are associated with decreased overall patient survival (42). Furthermore, small EVs from adriamycin-resistant cells contain HSP70 which directly targets mitochondria in recipient cells. In this way, HSP70 impairs mitochondrial function, promotes glycolysis, and induces therapy resistance in recipient cells (43). Taken together, these data suggest that therapy-resistant cells release EVs which promote glycolysis and therapy resistance in therapy-sensitive cells under hypoxic conditions. In this way, controlling EV content and/or glycolysis may represent a possible novel approach to target resistant tumor cells.

## EV Release in Response to Chemotherapy and Acquisition of Therapy Resistance

Chemotherapy is another factor that contributes to generating an inflammatory TME by increasing the production of inflammatory cytokines or modulating cellular components of the TME, including the immune system (12). To date, chemotherapy remains the most frequently employed treatment for cancer. However although initially effective in a large percentage of patients, relapse often occurs within a few years following the treatment and patients die due to the development of drug resistance (44). A wide range of molecular and cellular mechanisms have been identified as important in contributing to the development of chemoresistance: 1) increased rates of drug efflux; 2) activation of survival signaling and inactivation of death signaling pathways; 3) epigenetic changes and 4) effects of the local tumor microenvironment (6). In this context, inflammation of the TME enhanced by chemotherapy also can contribute to the failure of therapy (13). Moreover, this microenvironment can promote the release of EVs from tumor cells that contribute to therapy resistance. Indeed, several reports show that chemotherapeutic agents induce the biogenesis and release of EVs from tumor cells with pro-tumorigenic activity, including the ability to transfer chemoresistance (45–49).

For example, cisplatin and paclitaxel based chemotherapy is widely used as first-line therapy in several cancers and leads to a significant reduction in the tumor size (50–52). However, the use of cisplatin for the treatment of ovarian cancer (OC) promotes the release of EVs that induce drug resistance in bystander cells by modulating the p38 and JNK signaling pathways to increase cisplatin resistance (53). Furthermore, EVs released from chemosensitive bladder cancer cells, in particular the non-stem cancer cell (NSCCs) population, in response to cisplatin or gemcitabine, another chemotherapeutic agent, also promote therapy resistance and additionally favor cancer stem cell (CSC) survival in response to chemotherapy (54). A proteomic analysis of the EV cargo implicated the transfer from NSCCs to CSCs present in the TME of protein synthesis/degradation machinery components, which are critical for CSC survival, maintenance, and plasticity. Even though, the large majority of NSCCs die in response to chemotherapy, they release EVs containing ribosomal proteins that are taken up by CSCs and induce protein synthesis, aiding CSCs in adapting to the post-therapy TME, ultimately resulting in resistance and disease (54).

Chemotherapy with paclitaxel also modulates EV biogenesis, thereby contributing to therapy resistance in recipient cells. In breast cancer cells, treatment with paclitaxel induces the release of exosomes highly enriched in the protein Survivin, a member of the inhibitor of apoptosis (IAP) protein family that blocks cell death (55), and the transfer of these exosomes to breast cancer cells promotes cell survival in a Survivin-dependent manner (56). Recent studies show that paclitaxel and doxorubicin chemotherapy increases the levels of miR-378a-3p and miR-378d, microRNAs associated with chemoresistance, in EVs derived from patients and preclinical models. The uptake of such EVs by recipient breast cancer cell promotes cancer stemness and chemoresistance *via* enhanced EZH2/STAT3 signaling (57). Paclitaxel and doxorubicin also promote the secretion of EVs from breast cancer cells, which contain several microRNAs that target the transcription factor One Cut Homeobox 2 (ONECUT2), a protein involved in the induction of CSC-like properties that allows cancer cells to survive in response to cytotoxic treatment and therefore contributes to chemoresistance (58). Doxorubicin also has been described to promote the release of EVs by another mechanism. Cancer cells treated with Doxorubicin stimulate the secretion of EVs enriched in the protein ATP-binding cassette sub-family B member 1 (ABCB1), a transporter involved in promoting the efflux of chemotherapeutic drugs (59), by the upregulation of Rab8B and downregulation of Rab5 proteins. Moreover, these EVs transfer ABCB1 to sensitive cancer cells and confer a transient drug-resistant phenotype by downregulation of Rab5 in the recipient cell (46).

In pancreatic cancer cells, following treatment with gemcitabine the acquisition of chemoresistance mediated by EVs has been described. In response to drug treatment, exosomes transfer to neighboring cells superoxide dismutase 2 (SOD2) and catalase (CAT) transcripts, which encode ROS-detoxifying enzymes, that improve cell viability in response to the chemotherapy (60). Furthermore, downregulation of the gemcitabine-metabolizing enzyme, deoxycytidine kinase (DCK) is in part responsible of chemoresistance acquisition *via* an indirect mechanism involving the transfer of its targeting miRNA (miR-155). Indeed, when pancreatic cells stimulated with the exosomes containing miR-155 were treated with anti-miR-155 to block the effect, the cells became more sensitive to gemcitabine. These findings show that DCK downregulation mediated by exosomes from gemcitabine treated cells provides a survival advantage to gemcitabine-treated pancreatic cells (60). Thus, chemotherapy has two major EV-related effects, on the one hand increasing EV production and on the other hand including pro-tumorigenic cargos, which when transferred to sensitive cells promote chemoresistance (**Supplementary Table 2**).

## Macrophage-Derived Extracellular Vesicles in Cancer Drug Resistance

Tumor-associated macrophages (TAMs) are the major cellular component from the immune system in the TME (61) and key mediators of inflammation that contributes to many of the hallmarks of cancer (25). In fact, the high presence of TAMs in the tumor stroma is associated with tumor progression and poor prognosis, since they participate in tumor angiogenesis, matrix remodeling, invasion, metastasis, immunosuppression, and drug resistance (62–65).

As the main participants in the inflammatory response in the TME, macrophages mediate drug resistance in cancer cells through various molecular mechanisms. One of them involves the polarization of macrophages, whereby TAMs acquire characteristics similar to those of M2 macrophages. In breast cancer cells, SGLT1 overexpression drives glucose uptake and lactic acid secretion, which promotes macrophage polarization to M2-like TAMs that then activate the EGFR/PI3K/Akt/SGLT1 signaling pathway in the tumor cells to induce resistance to tamoxifen (66). Likewise, M2 macrophage polarization induces resistance to fluorouracil (5FU) treatment in gastric cancer cells by promoting cell survival *via* the PI3K/Akt/NF-κB pathway and inducing cell invasion through increasing the expression of integrin β3, FAK, and cofilin (67). Another report describes a similar mechanism whereby M2-polarized TAMs secrete CC chemokine ligand 2 [CCL2 also known as MCP-1)], which activates the PI3K/Akt/mTOR signaling pathway and promotes tamoxifen resistance in endocrine‐resistant breast cancer cells (68). Moreover, it has been observed that TAMs might be able to induce epithelial to mesenchymal transition (EMT) and consequently decrease sensitivity to the chemotherapeutic agent gemcitabine in pancreatic cancer cells (69). In addition, M2 macrophages induce the release of pyrimidine nucleosides, such as deoxycytidine, that confer resistance to gemcitabine in pancreatic cancer cells, by a mechanism of molecular competition at the level of drug uptake and metabolism (70).

However, the mechanisms responsible for cancer progression and drug resistance are currently being re-evaluated with the discovery of EVs as new players in this process. One of the principal mechanisms described is the exosomal transfer of miRNA from macrophages to tumor cells. For instance, it has been reported that TAM-derived EVs containing miR-365 induce resistance to gemcitabine in pancreatic adenocarcinoma cells, through a mechanism that involves an alteration in the metabolism of pyrimidine and an increase in cytidine-deaminase, the enzyme responsible for the inactivation of gemcitabine in humans (71). Similarly, EVs derived from a population of anti-inflammatory human macrophages contain proteins such as chitinase 3-like-1 and fibronectin, which decrease the sensitivity of pancreatic adenocarcinoma cells to gemcitabine by activating ERK (72). In oral squamous cell carcinoma (OSCC), EVs released by macrophages attenuate the susceptibility of cells to chemotherapeutic drugs, like 5-fluorouracil and cis-diaminedichloroplatinum, by activating the AKT/GSK−3β pathway (73). A similar mechanism has been reported in gastric cancer cells, where exosomal miR-21 is delivered by macrophages to cancer cells and prevents cisplatin-triggered apoptosis *via* inhibition of PTEN and subsequent activation of the PI3K/AKT pathway (74). Similarly, EVs shed from hypoxic macrophages transfer miR-223 to ovarian cancer cells to elicit a chemoresistant phenotype through the down-regulation of PTEN and activation of PI3K/AKT (75). Finally, crosstalk between neuroblastoma cells and human monocytes induces resistance to cisplatin through two exosomal signaling pathways involving the miR-21/TLR8-NF-кB and miR-155/TERF1 pathways (76).

Interestingly, the EV-mediated crosstalk between cancer cells and macrophages is bidirectional. EVs derived from ovarian cancer cells abundantly express exosomal miR-1246, which confers resistance to paclitaxel through inhibition of Caveolin-1 (CAV-1) and increased levels of multidrug resistance protein 1 (MDR1). Furthermore, ovarian cancer cells can also transfer their exosomal miR-1246 selectively to M2-type macrophages, which then produce lower CAV-1 mRNA levels. These results suggest that TAMs may indirectly play an important role in drug resistance mechanisms (77). Additionally, umbilical cord blood-derived M1 macrophage exosomes could be employed as vehicles for the administration of drugs in the treatment of platinum-resistant ovarian cancer cells (78). Taken together, these observations identify macrophages as important players in contributing to drug resistance. Furthermore, they uncover multiple signaling pathways involving the interaction between TAMs and cancer cells, whereby the pathway of choice appears to vary depending on the type of cancer cell and antitumor therapy (**Supplementary Table 3**). Importantly, they identify macrophage-derived EVs within the TME as promising molecular targets for restoring drug sensitivity, identifying potential drug response biomarkers and improving the efficacy of cancer therapies.

## Adipocyte-Derived Extracellular Vesicles in Drug Resistance

Obesity-associated adipose tissue dysfunction is characterized by several local and systemic changes, such as elevated levels of pro-inflammatory factors, sex hormones, lipid metabolites and altered levels of adipokines, which are implicated in carcinogenesis, tumor progression, metastasis, and alterations in therapy responses (79).

Several studies have reported on the mechanisms by which adipocytes contribute to resistance to anticancer drugs. For instance, adipocytes induce FABP4 expression by promoting metastasis and mediating Carboplatin resistance in ovarian cancer cells. Alternatively, the inhibition of FABP4 leads to increased levels of DNA demethylation, impairs metastasis and sensitizes cancer cells to Carboplatin chemotherapy (80). Also, adipocyte-conditioned medium reduces the sensitivity of HER2+ breast cancer cells to the cytotoxic activity of Lapatinib and other tyrosine kinase inhibitors. Soluble factors released from adipocyte lipolysis are likely to be responsible for the reduced activity of Lapatinib on breast cancer cells exposed to the adipocyte-conditioned medium (81). Similarly, it has been reported that the conditioned media from adipocytes contribute to the resistance of melanoma cells to chemotherapeutic drugs (Cisplatin and Docetaxel) and therapeutic agents targeting the PI3K/Akt and MEK/ERK pathways (82). Along the same line, another study shows that adipocytes secrete soluble factors that increase resistance to chemotherapeutic drugs in ovarian cancer cells by activating the Akt pathway (83). Interestingly, adipocytes reportedly protect acute lymphoblastic leukemia (ALL) cells from chemotherapy drugs (84) and even sequester and metabolize Daunorubicin (DNR) to an inactive form, allowing nearby ALL cells to avoid DNR-induced cytotoxicity (85).

While the effects of adipocytes are well-documented, studies implicating adipocyte-derived EVs in drug resistance are limited. One study reported that EVs from cancer-associated adipocytes (CAAs) delivered the miR21 to ovarian cancer cells, where it suppresses apoptosis and induces Paclitaxel resistance, as well as an aggressive phenotype by binding directly to a novel target APAF1 (86). Also, crosstalk mediated by EVs between multiple myeloma (MM) cells and adipocytes has been described, whereby exosomal adipocyte LncRNAs contribute to MM therapy resistance and in turn, MM cells educate adipocytes through the EZH2/METTL7A/LncRNA axis (87). Finally, adipocytes confer a multidrug resistance phenotype to breast cancer cells by increasing the nuclear efflux of Doxorubicin (DOX) through a major vault protein (MVP)-dependent process and its expulsion from breast cancer cells *via* EVs (88). In summary (**Supplementary Table 4**), multiple mechanisms have been shown to be involved in adipocyte-mediated drug resistance in various cancers. However, less is known about the role of adipocyte-derived EVs in the mechanisms leading to drug resistance. One may anticipate that greater insight in this respect could contribute to the development of new strategies to prevent the development of drug resistance.

## EVs From CAFs in Cancer Therapy Resistance

Cancer-associated fibroblasts (CAFs) are naturally resistant to cancer therapy. Moreover, CAFs contribute to therapy resistance through their crosstalk with cancer cells in several ways. Soluble compounds, such as cytokines and growth factors, have been implicated in this type of intercellular communication. For instance, therapy resistant CAFs produce and secrete IL-6, which has paracrine effects in cancer cells, thereby promoting chemotherapy resistance. Indeed, IL-6 upregulation is associated with poor prognosis in gastric cancer patients (89). IL-6 activates the JAK1/STAT3 signaling pathway in cancer cells (89, 90), and increases MDM2 expression, thereby promoting p53 polyubiquitination and degradation, which enhances cancer cell survival following drug treatment (91). In addition, IFN-β1 expression by CAFs is induced after the chemotherapy, leading to paracrine effects in breast cancer cells. The expression of IFN-β1 is related to reduce survival after chemotherapy (92). Furthermore, IL-1, in association with TGF-β1, induces the recruitment and transformation of normal fibroblasts to CAFs, which subsequently secrete pro-inflammatory factors that activate JAK/STAT and PI3K/Akt pathways in cancer cells, finally promoting therapy resistance (93). Moreover, patient-derived xenografts (PDX) resistant to cetuximab express higher levels of TGF-β1 in CAFs than xenografts sensitive to drug treatment (94). TGF-β1 secreted by CAFs upregulates the expression of ATF4 in cancer cells *via* the SMAD2/3 pathway. ATF4 promotes the expression of ABCC1 which favors the development of multiple drug resistance in cancer cells by extrusion of chemotherapy drugs (95). Also, CAFs secrete IGF-1 and HGF, as well as induce ANXA2 expression, which is required for CAF-induced EMT and therapy resistance (96). Also, CAFs secrete stromal cell-derived factor 1 (SDF-1 also known as CXCL12) which induces cancer cell drug resistance *via* a CXCR4, NF-κB and Bcl-xL-mediated signaling pathway (97). Finally, BDNF released from CAFs promotes therapy resistance *via* the TrkB/Keap1-Nrf2 pathway. Cancer cell-derived lactate upregulates BDNF expression in CAFs *via* the NF-κB pathway, thereby promoting a feedback amplification loop (98).

Soluble factors are however not the only components released by CAFs. Indeed, many molecules implicated in conferring drug resistance are transferred from CAFs to cancer cells in EVs. Moreover, there is strong evidence highlighting the relevance of EVs derived from CAFs in promoting cancer cell survival, proliferation, and subsequently drug resistance. Furthermore, the transfer of miRNAs in EVs from CAFs to cancer cells is commonly observed in connection with therapy resistance. Indeed, controlling the expression of pumilio homolog 2 protein (PUM2), an RNA-binding protein, appears to represent a novel mechanism to prevent therapy resistance. This protein is responsible for the packaging of miRNA-130a into exosomes, which are delivered from CAFs to lung cancer cells and promote cisplatin resistance (99). Another miRNA delivered by CAFs to cancer cells is miR-196a, which targets CDKN1B and ING5 in head and neck cancer cells and also confers cisplatin resistance (100). Moreover, gemcitabine resistant CAFs transfer miR-106b-containing EVs to pancreatic cancer cells, thereby promoting therapy resistance by targeting TP53INP1 (101), also known to be implicated in inducing drug-resistance in GC and BC (102, 103). In OC, paclitaxel-resistant CAFs transfer miR-21 containing EVs to cancer cells targeting APAF1 and apoptosis, thereby promoting therapy resistance (86). The latter mechanism has also been shown to be relevant in melanoma (104). Another miRNA delivered from CAFs to cancer cells related with paclitaxel resistance is miR-148b-3p, which induces the PTEN/Wnt/β-catenin pathway (105). This signaling pathway is also targeted by miR-92a-3p-containing EVs from CAFs in chemoresistant colorectal cancer cells (106). Also, miR-24-3p is transferred from CAFs to colon cancer cells targeting CDX2 and HEPH and promoting methotrexate resistance (107). Finally, prostate cancer cells acquire therapy resistance after miR-423-5p transfer in EVs from CAFs, which activates the TGF-β signaling pathway and controls Gremlin-2 expression (108).

However, miRNAs are not the only molecules relevant in therapy resistance delivered from CAFs to cancer cells. EVs containing Annexin-6 are transferred from CAFs to gastric cancer cells, thereby promoting therapy resistance though β1 Integrin/FAK-YAP activation (109). Moreover, lncRNA are delivered from CAFs to cancer cells. In fact, the lnc-RNA AFAP1-AS1 is present in CAF EVs and enhances the translation of ERBB2 mRNA by binding to AUF1, to induce the upregulation of HER-2 protein levels and subsequently trastuzumab resistance in breast cancer cells (110). Also, colorectal cancer associated lncRNA is transferred from CAFs to cancer cells through EVs and interacts with the mRNA stabilizing protein HuR (human antigen R) to increase β-catenin mRNA and protein levels, thereby promoting oxaliplatin resistance (111).

In summary (**Supplementary Table 5**), CAFs are resistant to therapy, and transfer proteins, miRNAs and lncRNAs in EVs to cancer cells. In doing so, CAFs induce therapy resistance. Thus, modulating either EV production by CAFs or their content could represent a novel therapeutic option for the treatment of non-sensitive tumors.

## CSC-Derived EVs in Therapy Resistance

In the TME, there are different types of cells that contribute to tumor progression, and specifically within tumors there is a small population with referred to as cancer stem cells (CSCs), which display the capacity of self-renewal, the ability to differentiate to other cell types and thereby to initiate, as well as maintain tumor growth (112). These cells are held responsible for generating drug resistance in many types of tumors because they display several properties that permit escaping from the consequences of chemotherapy. Moreover, they also can convert into many cell types associated with drug resistance, as mentioned previously (6, 112–114). Consistent with the relevance of the TME, CSCs are considered a component of this pro-inflammatory network because CSCs express different cytokine receptors, which bind to inflammatory cytokines, such as interleukin (IL)-1, IL-6, and IL-8, present in the TME (115). Since drug resistance is one of the main properties of CSCs, EVs released by these cells can transfer therapy resistance to sensitive tumor cells by delivering specific molecules that activate a drug resistance phenotype in the recipient cells.

For example, in a hepatocellular carcinoma (HCC) model, CSCs were found to release larger amounts of exosomes, a sub-type of EVs, in comparison with the non-CSC population of the tumor cells, and the secretion was mediated by Rab27a (116). Interestingly, the exosomes derived from the CSCs upregulate the expression of Nanog in recipient tumor cells and the acquisition of regorafenib resistance (116). To identify cells with CSC properties in the TME, several markers have been identified. A protein typically identified in several types of cancers is the transmembrane glycoprotein CD133 (117). For instance, Kang et al. reported that colon cancer cells release EVs containing CD133 in response to epidermal growth factor (EGF). In addition to activating the NF-κB signaling pathway, these EVs transfer the oncogenic protein KRAS to the recipient cells, thereby promoting the development of resistance against anti-EGF receptor (EGFR) drugs (118).

The CSCs are commonly found in hypoxic niches in tumors and hypoxia promotes CSC survival (119). In this context, Yin and colleagues observed that EVs derived from hypoxic glioma stem cells (GSCs) transfer temozolomide resistance to glioblastoma cells by delivering the miR-30b-3p, which targets RHOB to avoid apoptosis induced by the drug (120). Another study suggested that exosomes secreted by hypoxic glioma cells, which are enriched in CSCs, transfer the miR-301a and activate the Wnt/β-catenin signaling pathway by targeting TCEAL7 in glioblastoma cells, thereby promoting radiotherapy resistance (121).

A study in pancreatic cancer (PC) identified another miRNA responsible for therapy resistance mediated by CSC-EVs. Yang et al. reported that exosomes derived from pancreatic CSCs, which are resistant to gemcitabine, have high levels of miR-210. Transfer of this miRNA in exosomes to sensitive cells activates the mammalian target of rapamycin (mTOR) signaling pathway conferring resistance to gemcitabine-sensitive pancreatic cancer cells (122). In addition, CSC-EVs derived from OSCC contain miR-21-5p, another microRNA that activates mTOR. Such EV-mediated delivery of miR-21-5p and activation of the PI3K/mTOR/STAT3 signaling pathway in OSCC cells, leads to cisplatin resistance, increased clonogenicity and tumor sphere formation potential (123).

Another mechanism favoring the development of tumor cell resistance to anti-cancer therapies is activation of the EMT, because cells which activate this process acquire CSC properties (124). In this context, the role of exosomes as regulators of EMT has been investigated in many studies (125). Thus, by triggering this mechanism in recipient tumor cells, CSC-EVs also could transfer resistance to therapy. For example, it has been reported that miR-155 is an important regulator of EMT (126). Therefore, horizontal transfer of this miRNA mediated by EVs could confer resistance to therapy. Santos et al. demonstrated that exosomes derived from breast CSCs contain high levels of miR-155, and transfer of this miRNA to sensitive breast cancer cells reduces c/EBP-β activity, downregulate TGF-β and targets directly FOXO3a genes, resulting in the activation of EMT and acquisition of a chemoresistance phenotype against doxorubicin- and paclitaxel (127). In glioblastoma there is subtype of GSC called proneural (PN)-GSC and a more aggressive subtype called mesenchymal (MES)-GSC which display increased radio and chemoresistance. EVs derived from such MES-GSC cells increase stemness of normal PN cells, as well as therapeutic resistance to temozolomide, by inducing EMT through activation of the NF-κB/STAT3 signaling axis (128). Another example in which EMT is triggered by exposure to CSC-EVs has been reported for colon CSC-derived exosomes. These EVs contain Claudin-7, which induces EMT in low metastatic recipient cells, and likely also therapy resistance (129). Like CD133 in pancreatic cancer, CD44v6 is a marker of CSCs that promotes EV secretion. The transfer of such exosomes promotes resistance to apoptosis, as well as EMT in recipient cells by G protein-coupled receptor (GPCR) and integrin activation, transcription of EMT factors, and reduction of miRNA which target mRNAs from genes that contribute to self-renewal potential and migratory activity (130).

Finally, therapy resistance can be promoted indirectly by modulating the TME (131). CSC-derived EVs potentially modify the phenotype of many different types of cells in the TME and contribute thereby to therapy resistance. For instance, EVs liberated by renal CSCs promote *in vitro* the formation of capillary-like structures in matrigel (a proxy for vasculogenesis) and prevent doxorubicin-induced apoptosis in endothelial cells, which are required for tumor growth (132). In summary (**Supplementary Table 6**), CSCs display intrinsic properties that permit escaping from different types of anti-cancer treatments. Moreover, and quite importantly, they can transfer these properties *via* EVs to different cells present in the TME, which thereby become therapy resistant and this contributes to tumor progression.

## EVs in Antibody-Based Cancer Therapy Resistance

Several soluble pro-inflammatory factors released from cellular components of the TME activate signaling pathways in target cells that contribute to the tumor progression. Therefore, different therapies which block the interaction between such soluble factors and their receptors in cells have been developed. Antibody-based cancer therapy is one of the technologies used to block such interactions. The antibodies either bind specifically to the soluble factor neutralizing its effect or can target the surface receptor of the soluble factor and block its interaction with the ligand, therefore precluding triggering pro-tumorigenic signals (133). Among the different antibody-based cancer therapies, antibodies are commonly employed which block signaling pathways that promote development of the pro-inflammatory TME, such as those against vascular endothelial growth factor (VEGF), epidermal growth factor receptor (EGFR) or human epidermal growth factor receptor 2 (HER2) (134). Unfortunately, although antibody-based cancer therapy has proven to be successful, some patients also develop resistance to these types of treatment by different mechanisms (135, 136).

In this context, there is evidence demonstrating that EVs also participate in the development of resistance to antibody-based cancer therapy (**Supplementary Table 7**). One example is the antibody therapy against HER2, a receptor of the EGFR family, that promotes pro-tumorigenic properties by triggering different signaling pathways and is overexpressed in the 25-30% of BC (137, 138). HER2 triggers the IL-1α pro-inflammatory signaling pathway, which is important for maintenance of the CSC phenotype in HER2-positive breast cancers (139). Trastuzumab is a monoclonal antibody against HER2 which has yielded positive results in the treatment of metastatic breast cancer in patients with tumors overexpressing HER2 (140). Ciravolo et al. observed in the serum of HER2 breast cancer patients and in conditioned medium of HER2-overexpressing breast cancer cells the presence of exosomes containing functional HER2 protein. Importantly, release of these exosomes is modulated by the activation of HER2 in response to two different ligands (141). Moreover, these exosomes containing HER2 have the capability to bind trastuzumab *in vitro*, suggesting they act as antibody sponges and contributing therapy resistance by reducing trastuzumab availability for therapeutic purposes (141). Another way in which EVs can contribute to antibody-based cancer therapy resistance was observed using EGFR as a target. In cancer, EGFR activity drives tumorigenesis in different types of cancer since sustained activation triggers signaling pathways favoring cell survival, proliferation and migration that all contribute to tumor progression (142). Like HER2, the EGFR promotes CSC-like activity and tumor progression by activation of pro-inflammatory signaling (143). For this reason, the EGFR is considered a good candidate for targeted therapy. At least four EGFR-specific antibodies are used in clinical settings, namely cetuximab, panitumumab, nimotuzumab and necitumumab (144). Unfortunately, here too cases have been reported where cancer patients develop resistance to the treatments involving these antibodies (145, 146). For instance, OSCC is one of the cancers typically treated with the anti-EGFR antibody cetuximab; however resistance to this drug has been observed, since OSCC release EVs containing EGFR in response to EGF or cetuximab. These EVs can bind to and sequester cetuximab providing thereby a mechanism to explain how resistance against therapeutic anti-EGFR antibodies can develop (147).

Tumor progression depends on multiple cellular process, but angiogenesis is considered one of the most important due to its relevance in supplying the primary tumor with oxygen and nutrients that promote growth, facilitate the dissemination of tumor cells to generate metastasis, and contribute to inflammation in cancer (148, 149). Therefore pro-angiogenic factors are excellent therapeutic targets for antibody-based cancer therapy. Particularly VEGF and its receptor are the most common angiogenic signaling molecules used as targets in the treatment of several types of cancer (150). Again, although such antibody-based treatments have a favorable impact on cancer patient survival, the effects are not permanent due to the development of resistance (151). In this context, EVs also contribute to the acquisition of resistance to therapies that target VEGF signaling. Bevacizumab is a humanized monoclonal anti-VEGF antibody used to treat several solid tumors (152). In glioblastoma, bevacizumab is used as a therapeutic agent to block angiogenesis (153). However, glioblastoma cells have the ability to internalize and sort the antibody to the surface of the EVs produced by these cells, as well as change the proteome of the EVs released, which in combination is associated with therapeutic resistance (154). VEGF also can be sorted to the surface of tumor cell EVs. An isoform of VEGF (VEGF_189_) is preferentially found on the surface of the EVs, where in conjunction with heparin, it can sequester bevacizumab, thereby contributing to therapy resistance (155). Recently, other EV-specific mechanisms relating to anti-VEGF therapy resistance have been described. VEGF produced by tumor cells is captured by the protein CD63 present on the surface of EVs and packaged within the EVs in response to anti-VEGF therapy. This process reduces the accessibility of bevacizumab to the VEGF (156). On the other hand, the VEGF loaded inside the EVs can be internalized by endothelial cells where it triggers intracellular signaling events that promote angiogenesis and therefore generate resistance to the anti-VEGF therapy (156).

## Conclusions

**Figure 1:**
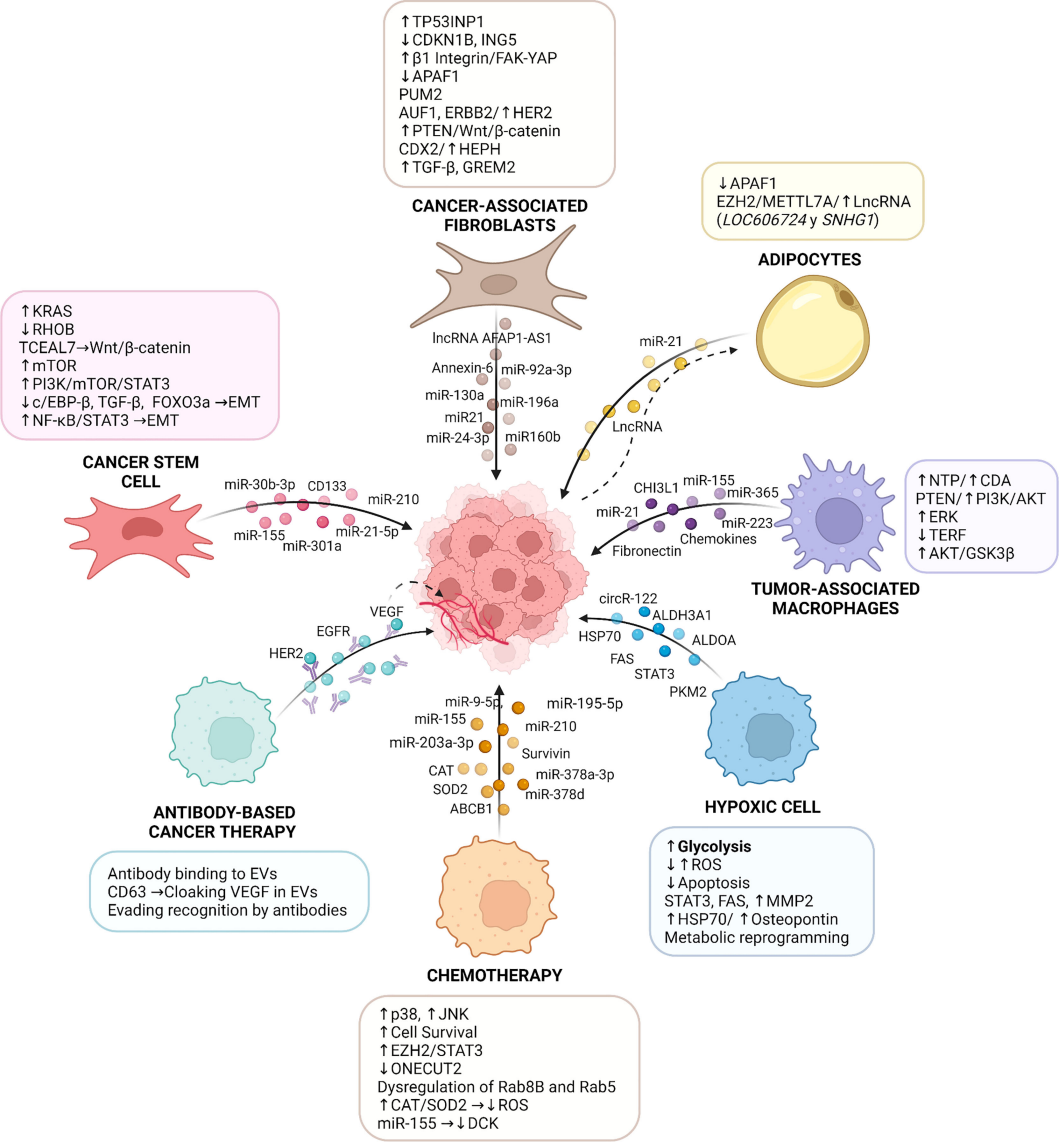
Role of Extracellular Vesicles in Cancer Therapy Resistance. The tumor microenvironment (TME) is involved in the initiation and maintenance of resistance to therapies by multiple molecular mechanisms. Specifically, extracellular vesicles derived from TME cells (e.g., cancer-associated fibroblasts, endothelial cells, cancer stem cells, immune cells, and adipocytes) transfer a variety of bioactive molecules, including mRNA, miR, lncRNA and proteins, which all play important roles in the communication between stromal components and tumor cells, activating in the latter signaling pathways that lead to cancer therapy resistance. In addition, resistance to therapy can be triggered by an inflammatory TME caused by conditions, such as hypoxia, or following chemotherapy, which modulate the content and release of EVs and alter the responses of tumor cells to cancer therapies. EVs also participate in resistance to antibody-based cancer therapy where cancer-derived extracellular vesicles package elevated amounts of validated targets for cancer treatment (e.g., VEGF, EGFR, and HER2), which are recognized by therapeutic antibodies and compromise the response of cancer cells to these therapies. TP53INP1: tumor protein p53 inducible nuclear protein 1; CDKN1B: cyclin-dependent kinase inhibitor 1B; ING5: inhibitor of growth family 5; FAK: focal adhesion kinase; YAP: yes-associated protein 1; PUM2: pumilio homolog 2 protein; AUF1: AU-binding factor 1; HER2: human epidermal growth factor receptor 2; CDX2: caudal‐related homeobox 2; HEPH: hephaestin; TGF-β: transforming growth factor β; GREM2: gremlin 2; APAF-1: apoptotic peptidase activator factor 1; RHOB: ras homolog family member B; TCEAL7: transcription elongation factor A-like 7; mTOR: mammalian target of rapamycin; PI3K: phosphoinositide-3-kinase; STAT3: signal transducer and activator of transcription 3; c/EBP-β: CCAAT enhancer binding protein-β; FOXO3a: forkhead box O3a; EMT: epithelial-mesenchymal transition; AFAP1-AS1: actin filament associated protein 1 antisense RNA 1; EZH2: enhancer of zeste homolog 2; METTL7A: methyltransferase like 7A; LncRNA: long noncoding RNA; SNHG1: small nucleolar RNA host gene 1; NTP: triphosphate-nucleotide; CDA: cytidine-deaminase; TERF: telomeric repeat-binding factor 1; GSK3β: glycogen synthase kinase 3 β; ROS: reactive oxygen species; FAS: fatty acid synthase; HSP70: heat shock 70 kDa protein; ONECUT2: factor One Cut Homeobox 2; ABCB1: ATP-binding cassette sub-family B member 1; CAT: catalase; SOD2: superoxide dismutase 2; DCK: deoxycytidine kinase; EGFR: epidermal growth factor receptor; VEGF: vascular endothelial growth factor; ALDH3A1: aldehyde dehydrogenase 3 family member A1; ALDOA: aldolase A; CHI3L1: chitinase 3-like-1. The figure was created with BioRender.com.

During the past decades our understanding of the mechanisms leading to therapy resistance has evolved from focusing exclusively on intrinsic properties of tumor cells to implicating also the inflammatory TME. Indeed, cells of the inflammatory TME are resistant to therapy and transfer this ability to tumor cells. EVs are relevant mediators of signaling between cells. In different contexts, EVs participate in physiological and pathological events. In cancer, EVs have been implicated in transformation, progression and metastasis, due to their ability to communicate between cancer cells and the tumor microenvironment. However, the role of EVs in transferring therapy resistance from stromal to tumor cells has only become apparent in more recent years. In this review, we summarized the studies describing the relevance of vesicles (generally defined as EVs following the ISEV guideline) in the development of therapy resistance following chemotherapy. In this context, EVs have been shown to transfer protein/miRNA/lncRNA cargos from the TME to tumor cells, to modulate survival, metabolism and EMT in these recipient cells (**Figure 1**).

After cancer therapy, the resulting inflammatory microenvironment contains tumor-resistant cells, hypoxic cells, CSCs, macrophages, adipocytes, and fibroblasts, which transfer EVs to treatment-sensitive cells and promote therapy resistance. Several proteins (such as STAT3, fibronectin, Survivin), miRNAs (such as miR21, miR155, miR210), LncRNAs and circRNAs are common cargos of EVs involved in conveying resistance. These cargos activate signaling pathways (such as PI3K/Akt, ERK, RAS, FAK) in tumor cells, thereby inducing changes in metabolism, survival, metastatic potential, and subsequently therapy resistance. Moreover, another direct mechanism involved in therapy resistance is the transfer of the protein ABCB1 in EVs from therapy-resistant to sensitive cells. Uptake of ABCB1 by recipient cells enhances drug efflux and the acquisition of resistance to the cancer treatment. In addition, EVs can act as sponges that sequester antibodies used in antibody-based cancer therapy. An example here is the recruitment of trastuzumab which reduces its effects on cancer cells (**Figure 1**).

Taken together, this review highlights the relevance of EVs in the acquisition of therapy resistance after the development of an inflammatory tumor microenvironment following cancer treatment. By summarizing this literature, we hope to encourage the search for novel cancer treatments that also consider controlling EV production in the TME.

## Author Contributions

LS, SS, BG-R, MV-G, and AQ organized the entire manuscript, wrote the draft, and revised the last version of the manuscript. Figure was designed by SS. All authors contributed to the article and approved the submitted version.

## Funding

This research was funded by FONDECYT grants 1210644 (AQ) and 1190928 (MV-G), FONDAP grant 15130011 (AQ), ANID/BASAL/FB210008 (MV-G), ANID FONDECYT postdoctoral fellowship 3190330 (LS), ANID PhD fellowship 21211248 (SS).

## Conflict of Interest

The authors declare that the research was conducted in the absence of any commercial or financial relationships that could be construed as a potential conflict of interest.

## Publisher’s Note

All claims expressed in this article are solely those of the authors and do not necessarily represent those of their affiliated organizations, or those of the publisher, the editors and the reviewers. Any product that may be evaluated in this article, or claim that may be made by its manufacturer, is not guaranteed or endorsed by the publisher.
