# Supplementary material for: Role of the Pro-Inflammatory Tumor Microenvironment in Extracellular Vesicle-Mediated Transfer of Therapy Resistance
Source: Front Oncol. 2022 May 11;12:897205. doi: 10.3389/fonc.2022.897205 (PMC9130576; doi:10.3389/fonc.2022.897205)
Supplement: Supplementary file 1 [file Table_1.pdf]

Supplementary Tables

TABLE 1 | EVs in therapy resistance induced by hypoxia

Therapy Resistance	Cancer Type	EVs content	Mechanism	Reference
Cisplatin	Non-Small-Cell Lung cancer	PKM2	PKM2, glycolysis, ROS, apoptosis: Bcl2	(39)
Oxaliplatin	Colorectal cancer	circR-122	miR-122, PKM2, glycolysis	(40)
Irradiation	Lung cancer	ALDOA, ALDH3A1	ALDOA, ALDH3A1, glycolysis	(41)
Cisplatin	Ovarian cancer	STAT3 and FAS	STAT3, FAS, MMP2	(38)
Radio(chemo)therapy	Non-Small-Cell Lung Cancer	HSP70	HSP70, Osteopontin	(42)
Adriamycin	Breast cancer	HSP70	HSP70, metabolic reprogramming	(43)

PKM2: pyruvate kinase M2; ROS: reactive oxygen species; Bcl2: B-cell lymphoma 2; circR: circular RNA; miR: microRNA; ALDOA: aldolase A; ALDH3A1: aldehyde dehydrogenase 3 family member A1; STAT3: signal transducer and activator of transcription 3; FAS: fatty acid synthase; MMP2: matrix metallopeptidase 2; HSP70: heat shock 70 kDa protein.

TABLE 2 | EVs in chemotherapy resistance

Therapy Resistance	Cancer Type	EVs content	Mechanism	Reference
Cisplatin	Ovarian cancer	-	P38/JNK	(53)
Gemcitabine	Bladder cancer	Proteins that regulate proteostasis	Non-stem cancer cells-derived EVs → increasing of CSCs aggressiveness	(54)
Paclitaxel	Breast cancer	Survivin	Increasing of cell survival mediated by survivin	(56)
Paclitaxel and doxorubicin	Breast cancer	miR-378a-3p and miR-378d	EZH2/STAT3	(57)
Docetaxel and doxorubicin	Breast cancer	miR-9-5p, miR-195-5p, and miR-203a-3p	ONECUT2 → expression of stemness-associated genes.	(58)
Doxorubicin	Non-small lung carcinoma	ABCB1	Promotion of drug efflux mediated by ABCB1	(46)
Gemcitabine	Pancreatic cancer	miR-155, CAT, SOD2	SOD, CAT → decreasing of ROS levels → Higher cell viability<br>miR-155 → downregulation of DCK.	(60)

JNK: c-Jun N-terminal kinase; EZH2: enhancer of zeste homolog 2; STAT3: signal transducer and activator of transcription 3; ONECUT2: factor One Cut Homeobox 2; ABCB1: ATP-binding cassette sub-family B member 1; CAT: catalase; SOD2: superoxide dismutase 2; DCK: deoxycytidine kinase.

TABLE 3 | Macrophage-derived EVs in therapy resistance

Therapy Resistance	Cancer Type	EVs content	Mechanism	Reference
Gemcitabine	Pancreatic ductal adenocarcinoma	miR-365	miR-365, NTP, CDA, Gemcitabine deamination	(71)
Gemcitabine	Pancreatic ductal adenocarcinoma	chitinase 3-like-1 and fibronectin	ERK	(72)
5-FU and CDDP	Oral squamous cell carcinoma	Chemokines (CCL2, CXCL8, etc.)	AKT/GSK-3β	(73)
Cisplatin	Gastric cancer	miR-21	miR-21/PTEN-PI3K/AKT	(74)
Cisplatin	Ovarian cancer	miR-223	miR-223/PTEN-PI3K/AKT	(75)
Cisplatin	Neuroblastoma	miR-21 and miR-155	miR-21/TLR8-NF-κB and miR-155/TERF	(76)

5-FU: 5-fluorouracil; CDDP: cisplatin; NTP: triphosphate-nucleotide; CDA: cytidine-deaminase; CCL2: C-C motif chemokine ligand 2; CXCL8: CXC motif chemokine ligand 8; GSK-3β: glycogen synthase kinase 3 β; PTEN: phosphatase and tensin homolog; PI3K: phosphoinositide-3-kinase; TLR8: toll-like receptor 8; NF-κB: Nuclear factor-kappa B; TERF: telomeric repeat-binding factor 1.

TABLE 4 | Adipocyte-derived EVs in therapy resistance

Therapy Resistance	Cancer Type	EVs content	Mechanism	Reference
Paclitaxel	Ovarian cancer	miR-21	miR21/APAF1	(86)
Bortezomib, Melphalan, Carfilzomib	Multiple myeloma	LOC606724 or SNHG1	EZH2/METTL7A/LncRNA axis.	(87)
Doxorubicin (with cross-resistance to paclitaxel and 5-FU)	Breast cancer	-	Major vault protein	(88)

APAF1: apoptotic peptidase activator factor 1; LOC606724: coronin 1A pseudogene; SNHG1: small nucleolar RNA host gene 1; EZH2: enhancer of zeste homolog 2; METTL7A: methyltransferase like 7A; LncRNA: long noncoding RNA.

TABLE 5 | CAF-derived EVs in therapy resistance

Therapy Resistance	Cancer Type	EVs content	Mechanism	Reference
Gemcitabine	Pancreatic cancer	miR-106b	TP53INP1	(101)
Cisplatin	Head and neck cancer	miR-196a	CDKN1B and ING5	(100)
Cisplatin	Gastric cancer	Annexin A6	β1 Integrin, FAK-YAP	(109)
Cisplatin	Non-small cell lung cancer	miR-130a	PUM2, miRNA-130a	(99)
Paclitaxel	Ovarian cancer	miR21	APAF1	(86)
Trastuzumab	Breast cancer	lncRNA AFAP1-AS1	AUF1, ERBB2 / HER2	(110)
Oxaliplatin	Colorectal cancer	colorectal cancer-associated lncRNA	mRNA stabilizing protein HuR → β-catenin	(111)
5-FU/L-OHP	Colorectal cancer	miR-92a-3p	miR-92a-3p, Wnt/β-catenin, FBXW7 and MOAP1	(106)
Methotrexate	Colon cancer	miR-24-3p	miR-24-3p, CDX2/HEPH	(107)
Doxorubicin and paclitaxel	Bladder cancer	miR-148b-3p	PTEN, Wnt/β-catenin	(105)
Taxane	Prostate cancer	miR-423-5p	miR-423-5p → TGF-β, GREM2	(108)

L-OHP: oxaliplatin; TP53INP1: tumor protein p53 inducible nuclear protein 1; CDKN1B: cyclin-dependent kinase inhibitor 1B; ING5: inhibitor of growth family 5; FAK: focal adhesion kinase; YAP: yes-associated protein 1; PUM2: pumilio homolog 2 protein; APAF1: apoptotic peptidase activator factor 1; AFAP1-AS1: actin filament associated protein 1 antisense RNA 1; AUF: AU-binding factor 1; HER2: human epidermal growth factor receptor 2; HuR: human antigen R; FBXW7: F-box and WD repeat domain containing 7; MOAP1: modulator of apoptosis 1; CDX2: caudal-related homeobox 2; HEPH: hephaestin; PTEN: phosphatase and tensin homolog; TGF-β: transforming growth factor β; GREM2: gremlin 2.

TABLE 6 | CSC-derived EVs in therapy resistance

Therapy Resistance	Cancer Type	EVs content	Mechanism	Reference
Regorafenib	Hepatocellular carcinoma	-	CSCs-EVs → Nanog expression in non-CSC and drug resistance	(116)
Anti-EGF	Colon cancer	CD133; KRAS	KRAS	(118)
Temozolomide	Glioblastoma	miR-30b-3p	RHOB	(120)
Radiotherapy	Glioblastoma	miR-301a	miR-301a →TCEAL7 → Wnt/β-catenin	(121)
Gemcitabine	Pancreatic cancer	miR-210	miR-210 → Rapamycin	(122)
Cisplatin	Oral squamous cell carcinoma	miR-21-5p	PI3K/mTOR/STAT3	(123)
Doxorubicin and paclitaxel	Breast cancer	miR-155	miR-155 targets FOXO3a genes and promotes downregulation of TGF-β, activity decreasing of c/EBP-β → EMT activation	(127)
Temozolomide	Glioblastoma	-	(MES)-GSC-EVs →stemness increasing in (PN)-GSC<br>EMT activation via NF-κB/STAT3	(128)
Cisplatin	Colon cancer	Claudin-7	Claudin-7 → EMT activation	(129)
Apoptosis resistance	Pancreatic cancer	CD44v6	EMT activation via GPCR; apoptosis resistance	(130)
Doxorubicin	Renal cancer	-	CSCs-derived EVs → apoptosis inhibition; formation of capillary-structures	(132)

RHOB: ras homolog family member B; TCEAL7: transcription elongation factor A-like 7; PI3K: phosphoinositide-3-kinase; mTOR: mammalian target of rapamycin; STAT3: signal transducer and activator of transcription 3; FOXO3a: forkhead box O3a; TGF-β: transforming growth factor β; c/EBP-β: CCAAT enhancer-binding protein-β; EMT: epithelial-mesenchymal transition; (MES)-GSC: mesenchymal glioma stem cells; (PN)-GSC: proneural glioma stem cells; GPCR: G protein-coupled receptors.

TABLE 7 | EVs in antibody-based cancer therapy resistance

Therapy Resistance	Cancer Type	EVs content	Mechanism	Reference
Trastuzumab	Breast cancer	HER2	HER2-positive EVs bind trastuzumab, decreasing drug levels for a therapeutic response.	(141)
Cetuximab	Oral squamous cell carcinoma	EGFR	EGFR-containing EVs sequester cetuximab sequestering	(147)
Bevacizumab	Glioblastoma	Bevacizumab	Bevacizumab is internalized by EVs	(154)
Bevacizumab	Ovarian, colorectal, and renal cancer	VEGF	VEGF on the surface of EVS, in conjunction with heparin sequester bevacizumab	(155)
Bevacizumab	Ovarian cancer	CD63	VEGF is captured by CD63 present on EVs surface → induction of angiogenesis	(156)

HER2: human epidermal growth factor receptor 2; EGFR: epidermal growth factor receptor; VEGF: vascular endothelial growth factor.
